# Clustering of dystonia in some pedigrees with autosomal dominant essential tremor suggests the existence of a distinct subtype of essential tremor

**DOI:** 10.1186/1471-2377-10-66

**Published:** 2010-07-29

**Authors:** Peter Hedera, Fenna T Phibbs, John Y Fang, Michael K Cooper, P David Charles, Thomas L Davis

**Affiliations:** 1 Department of Neurology, Vanderbilt University, 465 21st Avenue South, Nashville, TN, 37232- 8552, USA

## Abstract

**Background:**

There is an ongoing debate whether essential tremor (ET) represents a monosymptomatic disorder or other neurologic symptoms are compatible with the diagnosis of ET. Many patients with clinically definite ET develop dystonia. It remains unknown whether tremor associated with dystonia represent a subtype of ET. We hypothesized that ET with dystonia represents a distinct subtype of ET.

**Methods:**

We studied patients diagnosed with familial ET and dystonia. We included only those patients whose first-degree relatives met diagnostic criteria for ET or dystonia with tremor. This cohort was ascertained for the presence of focal, segmental, multifocal, hemidystonia or generalized dystonia, and ET.

**Results:**

We included 463 patients from 97 kindreds with autosomal dominant mode of inheritance (AD), defined by the vertical transmission of the disease. ET was the predominant phenotype in every ascertained family and each was phenotypically classified as AD ET. "Pure" ET was present in 365 individuals. Focal or segmental dystonia was present in 98 of the 463 patients; 87 of the 98 patients had ET associated with dystonia, one had dystonic tremor and ten had isolated dystonia. The age of onset and tremor severity did not differ between patients with "pure" ET and ET associated with dystonia. We did not observe a random distribution of dystonia in AD ET pedigrees and all patients with dystonia associated with ET were clustered in 28% of all included pedigrees (27/97, p < 0.001).

**Conclusions:**

Our results suggest that familial ET associated with dystonia may represent a distinct subtype of ET.

## Background

Essential tremor (ET) is the most common movement disorder, yet many uncertainties persist regarding its cause and clinical presentations [1]. Bilateral postural and kinetic arm tremor without significant asymmetry and the absence of additional neurologic abnormalities are the most commonly accepted diagnostic criteria for ET. There is an ongoing debate whether ET is truly a monosymptomatic disorder because many patients with otherwise typical ET develop dystonia in other body parts that are not affected by tremor [2,3]. It remains unknown whether postural and kinetic tremor associated with dystonia represents a subtype of ET. Alternatively, it may be a different form of tremor, unrelated to ET, because it has also been suggested that postural tremor seen in patients with cervical dystonia has a different pathophysiology than ET [4,5]. However, cervical dystonia, blepharospasm, and spasmodic dysphonia are commonly observed in patients with postural and kinetic tremor, which is clinically undistinguishable from typical ET [6,7]. This tremor needs to be differentiated from dystonic tremor, where dystonic posturing and tremor affect the same body part [8].

Previous reports of the coexistence of tremor and dystonia do not determine whether these two movement disorders associate randomly or whether shared susceptibility factors may explain their overlap. We hypothesized that only a subset of patients with familial ET develop additional signs of dystonia, suggesting that different genetic susceptibility factors play role in the development of "pure" ET and ET with dystonia. In order to demonstrate the presence of putative genetic susceptibility factors and to minimize the possibility of tremor phenocopies, we analyzed the distribution of dystonia in kindreds with autosomal dominant (AD) ET, or AD dystonia associated with tremor.

## Methods

**Figure 1:**
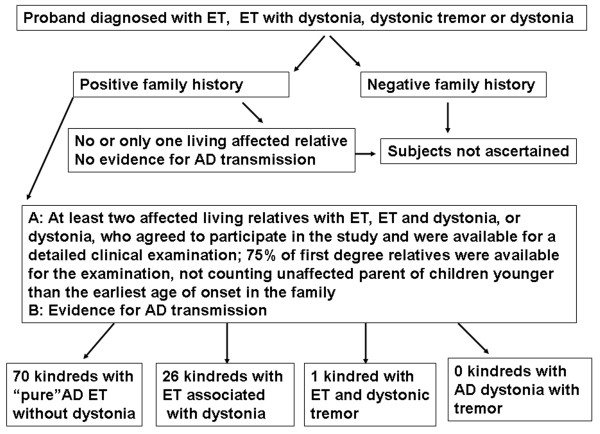
**The flow chart of inclusion criteria and ascertainment methods of kindreds, and the distribution of families with different types of tremor**.

Figure 1 depicts the flow chart for the ascertainment of included subjects. We recruited probands who met diagnostic criteria for ET or dystonia to minimize a possible bias towards families with a "pure" form of ET. Furthermore, the inclusion was limited to only patients who identified at least two living first-degree relatives affected by tremor and/or dystonia who also agreed to participate in this study; furthermore, we included only kindreds where we were able to evaluate at lest 75% of identified first degree relatives not counting the unaffected parent or children who were younger than the youngest age of onset in the studied pedigree. All probands were seen in the Movement Disorders Clinic at the Vanderbilt University Medical Center and were examined by movement disorder neurologists (PH, FP, JYF, MKC, PDC or TLD). All other recruited relatives were personally examined by PH; we did not include any subjects who were not personally examined. Probands who did not report family history of tremor or dystonia were not asked to recruit their apparently unaffected first-degree relatives. This investigation and all study procedures where approved in advance by the institutional review board for the protection of human subjects at Vanderbilt University Medical Center, and an informed consent was obtained from every enrolled subject.

Inclusion criteria for a positive family history encompassed evidence of vertical transmission for ET or dystonia consistent with an AD mode of inheritance. Thus, the minimal number of affected individuals (tremor, dystonia or both) was three in a single pedigree, and at least two of them suffered from the same condition, either tremor or dystonia.

Patients were evaluated for the presence of idiopathic dystonia and cases of presumed secondary dystonia were excluded. Dystonia was diagnosed and classified based on previously published criteria as focal, segmental, multifocal, hemidystonia and generalized [9]. Categories of focal dystonia included blepharospasm, cervical, laryngeal (spasmodic dysphonia), oromandibular, and isolated limb dystonia, including writer's cramp [9].

Postural and action tremor of the upper extremities was classified into three groups: "pure" or clinically definite ET, where no additional neurological problems were detected; ET associated with dystonia where dystonia did not involve the upper extremities affected by tremor; and dystonic tremor where tremor and dystonia coexisted in the same limb. Patients with a writer's cramp were classified as ET associated with dystonia because their dystonia was task-specific and not associated with dystonic posturing other than during writing. Clinically definite ET was defined as bilateral postural and kinetic tremor without any additional neurologic abnormalities including no dystonia or signs of hypokinetic-rigid syndrome, absent history of exposure to tremorogenic drugs before the onset of symptoms, and without history and examination suggestive of psychogenic tremor or sudden onset of tremor with a stepwise deterioration [1,10]. Tremor was graded using the Washington Heights-Inwood Genetic Study of Essential Tremor (WHIGET) scale [11,12]. Patients with tremor were also examined for the presence of a null point for their tremor, indicating arms positions which were associated with a tremor free state, and the regularity of action tremor, evaluated by Archimedes spiral drawing.

Demographic characteristics and tremor severity scores in ET patients with and without dystonia were compared using *t*-test. The distribution of dystonia among identified families was analyzed using chi-square tables.

## Results

**Figure 2:**
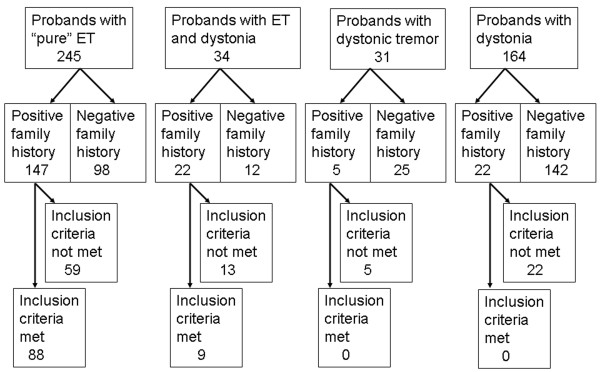
**The distribution of dystonia and tremor in screened patients**.

**Figure 3:**
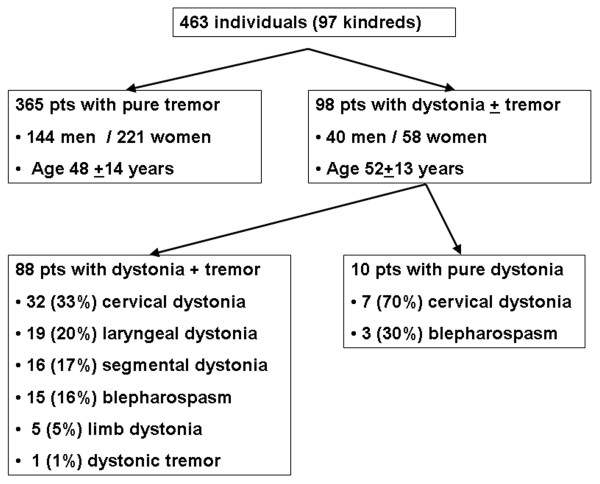
**The distribution of various types of dystonia in the selected cohort consisting of probands and their family members**.

We initially screened 474 patients who were diagnosed with ET, ET and dystonia, dystonic tremor or dystonia, and further investigated only those probands who reported a positive family history of tremor and/or dystonia (Figure 2). Ninety seven probands and 366 of their affected relatives met the inclusion, accounting for the study cohort of 463 individuals from 97 kindreds. Table 1 and Figure 3 show their clinical and demographic characteristics. Each of these kindreds had at least two individuals who met the diagnostic criteria for clinically definite ("pure") ET with signs of vertical transmission and we did not identify any families where the majority of affected individuals had signs of dystonia. Signs of dystonia were diagnosed in 98 individuals and 365 met diagnostic criteria for clinically definite ET with a "pure" postural and/or action tremor phenotype.

**Table 1:** Clinical characteristics of patients with "pure" ET and tremor associated with dystonia.

	"Pure" tremor	ET associated with dystonia	Isolated dystonia
Number of subjects	365 (144 M/221 F)	87 (35 M/52 F)	10 (5 M/5 F)

Average age (years)	48.32 ± 14.16	51.08 ± 10.80	49.45 ± 9.87

WHIGET scores	14.21 ± 6.78	12.81 ± 5.76	N/A

Age of Onset (years)	36.56 ± 17.65	32.78 ± 12.11	29.34 ± 19.45

Eighty seven patients with dystonia had a motor phenotype consistent with ET associated with dystonia (dystonia not affecting the upper extremities), one met diagnostic criteria for dystonic tremor affecting both arms, and ten had an isolated dystonia without postural or kinetic tremor (Figure 2). Among the patients with ET associated with dystonia, we detected only focal and segmental dystonia, while the other subtypes of dystonia were not present in patients who met the inclusion criteria. This subgroup of focal dystonias associated with ET consisted of 15 patients with blepharospasm (16%), 32 with cervical (33%), 19 with laryngeal (20%) and five with a limb dystonia (5%). Every patient with a focal limb dystonia had writer's cramp and we did not identify any individuals with other types of limb dystonia or a segmental limb dystonia associated with ET. Segmental dystonia was present in 16 patients (17%), and included only craniocervical dystonia consisting of a various combinations of blepharospasm, cervical, oromandibular, or laryngeal dystonia. The kindred with dystonic tremor consisted of five individuals with a "pure" ET and one individual had dystonic tremor affecting both upper extremities. We also identified 10 patients with a strong family history of ET who had an isolated dystonia without postural or kinetic tremor (Figure 2). Within this subgroup of patients three had blepharospasm and seven had cervical dystonia and the onset of dystonia was reported between 22 and 45 years (average 29.34 ± 19.45 years).

The patients with tremor and dystonia did not differ from patients with an isolated ET in the tremor severity or the age of onset (Table). Bilateral arm tremor was reported as an initial symptom in every patient with dystonia and ET with the exception of three individuals who reported dystonia (one blepharospasm and two cervical dystonia) as the first symptom of the disease. The subsequent onset of dystonia lagged from 8 to 12 years (average age 9.67 ± 2.11 years) after the beginning of tremor. The three individuals presenting with focal dystonia reported the emergence of bilateral arm tremor within five to seven years. Qualitative analysis of postural and action arm tremor between patients with a "pure" ET and tremor associated with dystonia did not identify any differences in tremor characteristics, and it showed regular, rhythmic oscillations without any irregular jerks or null points in both groups.

The distribution of patients with dystonia within the included kindreds was not random because only 27 families (28% of all included pedigrees) contained all patients diagnosed with dystonia (p < 0.001) (Figure 1, bottom row). Ten families had a single individual with dystonia, and the remainder had at least two. The highest number of affected individuals with ET and dystonia from single kindred was five. Each family with a solitary dystonia patient consisted of only three affected individuals, the minimum necessary to be included in this analysis, and the remaining two had always phenotype consistent with clinically definite ET. Thus, these families were classified as familial ET rather than familial dystonia based on the predominant motor phenotypes. Similar trend was observed when all patients with dystonia were analyzed, including probands without a formal family history (Table 2, p < 0.05).

**Table 2:** Proportion of patients with tremor and dytonia with and without positive family history (patients with dystonic tremor were considered as having dystonia).

	Positive family history of tremor	Negative family history of tremor
Positive family history of dystonia	98	27

Negative family history of dystonia	365	167

## Discussion

The coexistence of postural and/or kinetic tremor with dystonia is relatively common and is present in 4-55% patients with focal dystonia [8,13,14]. For patients with dystonia it remains controversial whether tremor in limbs not affected by dystonia represents ET or a different movement disorder. Postural tremor associated with dystonia has been previously described as irregular and occasionally associated with myoclonus [5]. The electromyographic patterns of voluntary ballistic movements of the wrist are different in patients with ET compared to those with cervical dystonia associated tremor [4]. One possible explanation is that the pathophysiology of the tremor in these two groups is fundamentally different. Alternatively, possible functional differences in tremor phenotype in patients with and without dystonia may also be due to different motor presentation of the same genetic process. Our analysis identified eligible kindreds with several members affected by "pure" (clinically definite ET without dystonia) ET, increasing the probability that individuals manifesting both tremor and focal dystonia share the same genetic risk factors as their first degree relatives with ET alone. The vast majority of affected patients had signs of otherwise typical ET without any additional abnormalities. Furthermore, there were no differences in clinical presentations of postural and kinetic tremor, such as age of onset or clinical severity between the patients with and without dystonia, supporting our phenotypic classification as ET in patients with additional neurologic signs.

It remains controversial whether "pure" ET, ET associated with focal dystonia, and dystonia with postural arm tremor are a part of a clinical continuum with variable clinical presentation of the same nosological entity, or they represent distinct diseases with different causes and pathophysiology. We did not identify any kindred with AD generalize dystonia associated with postural tremor, and we could only compare clinical characteristics between "pure" ET and ET associated with focal dystonia. Even though our study cannot provide a definitive answer, the coexistence of "pure" ET and ET associated with focal dystonia in the pedigrees with an AD mode of inheritance supports the role for putative shared genetic factors in their pathogenesis. Identification of genes causing ET and possible genetic modifiers will likely settle this question, and the collected pedigrees will hopefully facilitate this future work.

We detected the coexistence of ET and focal dystonia in 19% of patients in our cohort of AD ET. This is within the range of previously reported studies, which found the frequency of dystonia associated with ET varying from 0.6% to 47% of patients [6-8,15]. Undoubtedly, this broad range likely reflects diagnostic dilemmas and lack of universally accepted diagnostic criteria. Our results may partially underestimate the frequency of dystonia in ET patients because in order to detect possible genetic susceptibility to the development of dystonia, we only analyzed familial cases with at least three affected individuals. Interestingly, we did not identify any patients meeting the inclusion criteria who had signs of generalized or hemi-dystonia, suggesting that only focal dystonias are pathophysiologically related to ET. We have also identified ten patients with focal dystonia without postural or kinetic tremor of the upper limbs, suggesting that both tremor and dystonia may be the presenting motor manifestation in these kindreds. Thus, patients with focal dystonias who develop regular postural and/or kinetic tremor may be reclassified as a subtype of ET.

We ascertained kindreds through probands diagnosed with either ET or dystonia. The main reason to include probands with both of these conditions was to minimize a possible bias towards families with a "pure" form of ET if we analyzed only families previously diagnosed with AD ET. However, this approach may paradoxically introduce another bias because families with the coexistence of dystonia and tremor may be more likely to be included in our cohort. However, an increased proportion of families with coexisting dystonia would potentially diminish our finding that only a subset of kindreds with AD mode of inheritance develops dystonia. Thus, the detection of clustering of dystonia in one fourth of analyzed families in spite of a potential bias in our selection further supports our hypothesis.

We further investigated only those probands who indicated that their first degree relatives had either tremor or dystonia. It was not logistically feasible to examine their first-degree relatives to confirm the absence of neurologically affected individuals in these families. The sensitivity of family history obtained solely by reports of affected probands has very low sensitivity, with 27% sensitivity for the history of dystonia and only 16% for history of ET [16,17]. We cannot exclude a possibility that several kindreds for both AD ET and AD dystonia were missed, even though a poor sensitivity for both conditions would argue against the introduction of a systemic bias for ET pedigrees only. Similarly, we could not always completely ascertain the complete pedigrees with a confirmed family history and we used the minimal criterion of 75% of first-degree at risk relatives to be examined. However, given the poor sensitivity for the identification of both conditions, it is unlikely that this has introduced a particular bias towards the patients with ET.

## Conclusion

Our analysis identified a non-random distribution of ET associated with dystonia, which was not previously reported. These results support the notion that ET associated with dystonia may represent a distinct subtype of ET.

## Competing interests

The authors declare that they have no competing interests.

## Authors' contibutions

PH designed the study. PH, FTP, JYF, MKC, PDC and TLD examined research subjects and help them to recruit other family members. PH analyzed the data and drafted the manuscript. All authors read and approved the final manuscript.

## Pre-publication history

The pre-publication history for this paper can be accessed here:

http://www.biomedcentral.com/1471-2377/10/66/prepub
